# Probing the staying power of chemogenetics

**DOI:** 10.7554/eLife.109193

**Published:** 2025-10-13

**Authors:** Maria Puchik, Igor Kagan

**Affiliations:** 1 Decision and Awareness Group, Cognitive Neuroscience Laboratory, German Primate Center – Leibniz Institute for Primate Research Göttingen Germany

**Keywords:** primate research, DREADDs, monkey, PET, AAV, nonhuman primates, Rhesus macaque, Japanese macaque, Cynomolgus macaque

## Abstract

A study that monitored the expression and function of designer receptors called DREADDs in macaque monkeys for a period of three years demonstrates that they are effective in long-term studies of nonhuman primates.

**Related research article** Nagai Y, Hori Y, Inoue K, Hirabayashi T, Mimura K, Oyama K, Miyakawa N, Hori Y, Iwaoki H, Kumata K, Zhang MR, Takada M, Higuchi M, Minamimoto T. 2025. Longitudinal assessment of DREADD expression and efficacy in the monkey brain. *eLife* **14**:RP105815. doi: 10.7554/eLife.105815.

Our brain is arguably the most complex object in the known universe, containing tens of billions of individual neurons, each of which is connected to a number of other neurons. Understanding the brain means figuring out not only what each region does, but also how regions connect and interact. The most direct way to do this is to silence a region – or a connection – and observe the consequences. In the past, this approach relied on studies of patients with brain lesions, or on animal experiments in which lesions, pharmacological approaches or electrical microstimulation were used to suppress activity in specific regions of the brain (Figure 1A). However, it was not possible to target a specific population of neurons, or a specific connection between two regions of the brain (Klink et al., 2021).

**Figure 1.**
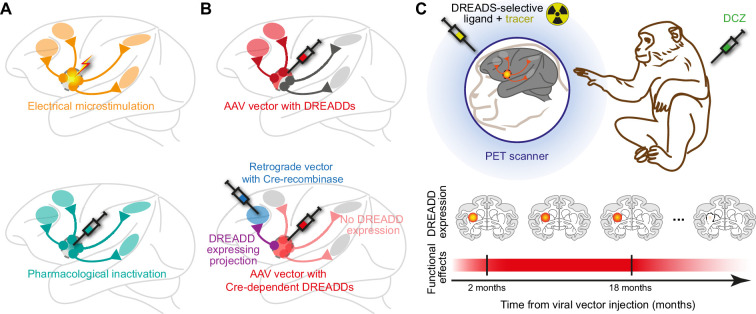
Chemogenetics makes it possible to manipulate specific populations of neurons within the brain. (**A**) Electrical microstimulation (top) and pharmacological inactivation (bottom) are non-selective approaches that perturb all projections from the target area (orange and cyan lines and interconnected brain regions). (**B**) Chemogenetic approaches use designer proteins called DREADDs to target specific populations of neurons. For example, DREADDs can be designed so that they only target projections from certain cell types using specific promoters (red lines; top), while leaving other projections unaffected (grey lines). An approach called dual-viral vector transduction (bottom) can be used to target a specific connection (purple line) between two brain regions (blue and red), while leaving other projections unaffected (light red lines). (**C**) Top: schematic illustration of PET imaging of DREADD expression, and functional assessment, in macaques. Bottom: Nagai et al. found that DREADD expression, and the corresponding functional (behavioural, neuronal, metabolic) effects of DREADDs, remained stable for up to 18 months, before starting to decline.

In recent years, a new powerful approach, chemogenetics, has made this possible. Chemogenetics relies on engineered receptors called DREADDs – which is short for designer receptors exclusively activated by designer drugs – that are inserted into specific neuronal populations with the help of viruses (Roth, 2016). The phrase “exclusively activated by designer drugs” means that these receptors are not affected by naturally occurring chemicals in the body: rather, DREADDs are engineered so that they can only be activated by compounds that have been designed for this purpose (Campbell and Marchant, 2018). This approach allows researchers to manipulate the neural activity of specific populations, or specific cell types, in a way that is highly controlled, non-invasive and reversible (Oguchi et al., 2021; Minamimoto et al., 2024; Figure 1B). Moreover, some DREADDs are designed to lead to an increase in neural activity, whereas others inhibit it.

The majority of chemogenetic experiments to date have been performed in rodents – the most common translational model in neuroscience – but rodents are much smaller than humans, and also differ greatly in brain structure and behavior. Nonhuman primates, such as macaques and marmosets, have brains that are more similar to human brains, and their cognition and behavioural repertoire more closely resemble our own, so studies of these species have an indispensable role in helping us to understand the human brain (Roelfsema and Treue, 2014; Raper and Galvan, 2022). However, the nature of primate research means that studies typically last for several years, which is much longer than the time needed for a typical rodent study. This raises a critical question: can chemogenetic techniques remain effective over such long periods? Now, in eLife, Yuji Nagai, Takafumi Minamimoto and colleagues at the National Institutes for Quantum Science and Technology and Kyoto University report the results of experiments with macaques which show that chemogenetic manipulations remain effective for over a year and a half (Nagai et al., 2025).

Nagai et al. employed a well-established viral vector – the adeno-associated virus – to deliver the DREADDs into specific neuronal populations, and then used an imaging technique called positron emission tomography (PET) to monitor the expression of the DREADDs over time. A PET scan detects radioactive tracers that have been injected into the organism being scanned. In their experiments Nagai et al. used a compound called [^11^C]DCZ, where [^11^C] indicates the presence of radioactive carbon-11, and DCZ is short for deschloroclozapine – a novel, highly selective ligand that binds to neurons expressing DREADDs (Nagai et al., 2020). By performing a PET scan after the viral delivery, it is possible to check that the DREADD is being expressed where it should be, and by carrying out follow-up scans, it is possible to see how long it remains expressed.

Nagai et al. used this approach to study a group of 15 animals across multiple studies. A subset of seven animals was scanned regularly for a period of 150 days to capture a short-term, zoomed-in view of expression dynamics, and 11 animals were scanned less frequently over a period of three years. The researchers found that expression peaked about 60 days after the injection, remained relatively stable for 1.5 years, and then gradually declined (Figure 1C). There were also some differences observed between the inhibitory DREADD (hM4Di) and the excitatory DREADD (hM3Dq). Crucially, the timing of the decline was unrelated to the number of DCZ injections, dispelling concerns about cumulative dosing effects over repeated experiments. Nagai et al. also showed that robust behavioural, neural and metabolic effects of DREADD activation persisted for roughly two years – and in some cases, even longer. Conversely, immunohistochemical brain analysis in one animal who lost expression and behavioural effects after three years confirmed the absence of DREADD receptors – but notably, revealed no evidence of neuronal loss.

These results, confirming the reliable and stable time course of chemogenetic effects, provide both a valuable foundation for future studies, and helpful methodological guidelines for the growing primate chemogenetics community. Beyond its value as a research tool, this and future long-term assessments of chemogenetics are also essential for potential clinical translation to humans.

However, the latest work also raises several important questions. One issue is whether the initial modest decline after the peak – seen in 150 day time course – can be mitigated. Another cautionary finding is that at least some of the protein tags used for histological detection may reduce DREADD expression, suggesting a possible trade-off between functional efficacy and detectability. It is also worth noting that the low spatial resolution of PET may limit its sensitivity for some applications, for instance when visualizing axonal projections. Moreover, not all laboratories have access to the equipment needed to perform PET imaging on macaques, so researchers in these labs will be forced to rely on already validated protocols, as derived from studies like Nagai et al., or on less direct measures such as resting-state functional imaging (Fujimoto et al., 2022). These and other challenges will define how primate chemogenetics evolves in the next crucial years.
